# Food Intake during School Lunch Is Better Explained by Objectively Measured Eating Behaviors than by Subjectively Rated Food Taste and Fullness: A Cross-Sectional Study

**DOI:** 10.3390/nu11030597

**Published:** 2019-03-12

**Authors:** Petter Fagerberg, Billy Langlet, Andrew Glossner, Ioannis Ioakimidis

**Affiliations:** 1 Innovative Use of Mobile Phones to Promote Physical Activity and Nutrition Across the Lifespan (the IMPACT) Research Group, Department of Biosciences and Nutrition, Karolinska Institutet, 14152 Stockholm, Sweden; billy.langlet@ki.se (B.L.); Ioannis.Ioakimidis@ki.se (I.I.); 2 Internationella Engelska Gymnasiet Södermalm, Allhelgonagatan 4, 11858 Stockholm, Sweden; Andrew.Glossner.sodermalm@engelska.se

**Keywords:** obesity, childhood, adolescents, cafeteria, sensory science, behavioral nutrition

## Abstract

School lunches contribute significantly to students’ food intake (FI) and are important to their long-term health. Objective quantification of FI is needed in this context. The primary aim of this study was to investigate how much eating rate (g/min), number of food additions, number of spoonfuls, change in fullness, food taste, body mass index (BMI), and sex explain variations in school lunch FI. The secondary aim was to assess the reliability of repeated FI measures. One hundred and three (60 females) students (15–18 years old) were monitored while eating lunch in their normal school canteen environment, following their usual school schedules. A subgroup of students (*n* = 50) participated in a repeated lunch (~3 months later). Linear regression was used to explain variations in FI. The reliability of repeated FI measurements was assessed by change in mean, coefficient of variation (CV), and intraclass correlation (ICC). The regression model was significant and explained 76.6% of the variation in FI. Eating rate was the strongest explanatory variable, followed by spoonfuls, sex, food additions, food taste, BMI, and change in fullness. All explanatory variables were significant in the model except BMI and change in fullness. No systematic bias was observed in FI (−7.5 g (95% CI = −43.1–28 g)) while individual students changed their FI from −417 to +349 g in the repeated meal (CV 26.1% (95% CI = 21.4–33.5%), ICC 0.74 (95% CI = 0.58–0.84)). The results highlight the importance of objective eating behaviors for explaining FI in a school lunch setting. Furthermore, our methods show promise for large-scale quantification of objectively measured FI and eating behaviors in schools.

## 1. Introduction

Ensuring healthy eating in schools has been argued to be an international human right [1]. Both under- and overconsumption of food can have serious health consequences, such as malnutrition and obesity, and can therefore be considered as unhealthy eating [2]. In the European context, around 115 million children and adolescents attend school for up to 13 years of their life [1]. The food environment in schools is of major importance for the health of students in the long-term, since ~40% of their food intake is expected to be consumed at school (throughout the day spread among numerous eating events) [3]. Furthermore, intervention studies to improve the dietary quality of school lunches have shown benefits with respect to school achievement, further demonstrating the importance of school lunches [4].

In Sweden, the law dictates that children and adolescents should be offered nutritious food free of charge during school lunches [5]. Swedish students have been reported to consume ~25% of their daily energy intake during school lunches [6], while 24% of Swedish high school students are overweight or obese [7,8]. However, data about school-based food intake are mainly collected through self-reported methodologies, known to be subject to systematic biases such as underreporting [9,10,11], especially among obese populations [12,13]. Therefore, the development and use of novel objective methods to measure food intake have been strongly encouraged [10]. 

An earlier effort to develop such objective methodology in the Swedish school context showed that children eating school lunch increased their speed of eating when eating in groups, compared to eating their meal by themselves [14]. The increased speed of eating was associated with increased food intake in the majority of boys and in some girls, with the remaining girls reducing their food intake [14]. Furthermore, Langlet and colleagues have previously reported on sex differences in eating behavior and food intake among 16–17-year-old high school students when eating school lunch [15]. Males ate more food on average vs. females, an observation that could be explained by larger bite sizes and higher eating rate. This observation is also supported by experimental studies conducted in laboratory settings on adults, showing increased food intake due to an experimentally induced increased eating rate [16]. Laboratory studies have also indicated associations among subjective measures of food liking, hunger, fullness, and food intake [17,18]. However, no objective studies have been conducted in a school lunch context to investigate these relationships.

Due to the importance of healthy eating among students and the fact that no objective studies have been conducted in a school setting with the aim to explain variations in food intake, the primary aim of this study was to investigate how much (i) eating rate, (ii) number of food additions, (iii) number of spoonfuls, (iv) change in fullness, (v) food taste, (vi) body mass index (BMI), and (vii) sex explain variations in food intake among high school students during a school lunch. The secondary aim was to assess the reliability of food intake for the same individuals across two school lunches served in the same environment with opportunity for identical food item selection. Our hypothesis was that objectively measured eating behaviors would have more explanatory power than subjectively measured food taste and change in fullness. The current study is an important effort to provide more accurate and valid measures of food intake among high school students with the possibility to identify potential targets for future school-based interventions against childhood obesity.

## 2. Materials and Methods

### 2.1. Study Design

A cross-sectional study design was used to explain the variation in food intake (primary aim) and a within-subject repeated-measures study design was used to evaluate the reliability of food intake measurements (secondary aim). 

### 2.2. Setting

**Figure 1:**
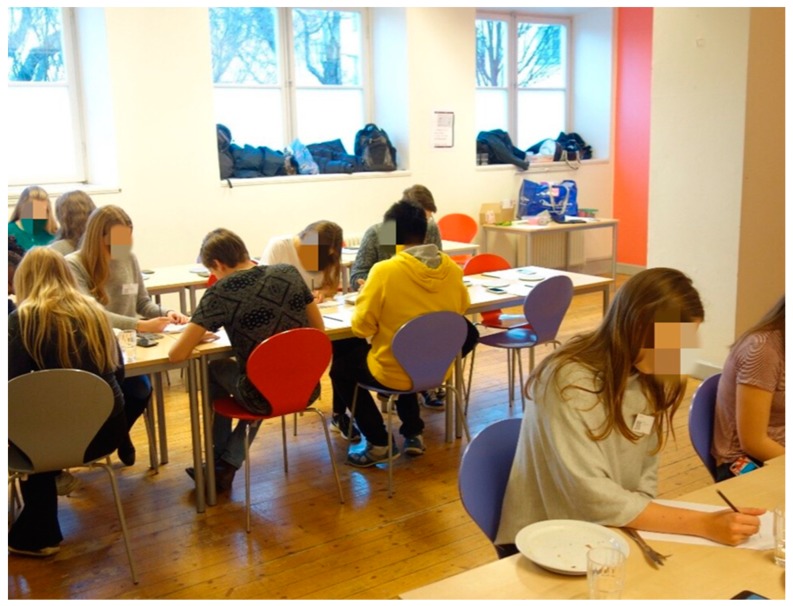
The lunchroom where the participating students ate their lunches.

All the school lunches were measured in a high school in central Stockholm, Sweden, with approximately 60% of students being female. Students were attending morning classes during data collection. During the scheduled lunchtime (either 11:30 AM or 12:30 PM), students were eating school lunch in their normal school canteen environment (see Figure 1). 

Normally, students have 25 min to collect and eat their food and the school lunch routine was strictly kept during the current study. Furthermore, the study was part of a technological EU project (SPLENDID) with the intention to develop a personalized guide for eating and activity behavior for the prevention of obesity and eating disorders [19,20]. 

### 2.3. Participants

**Figure 2:**
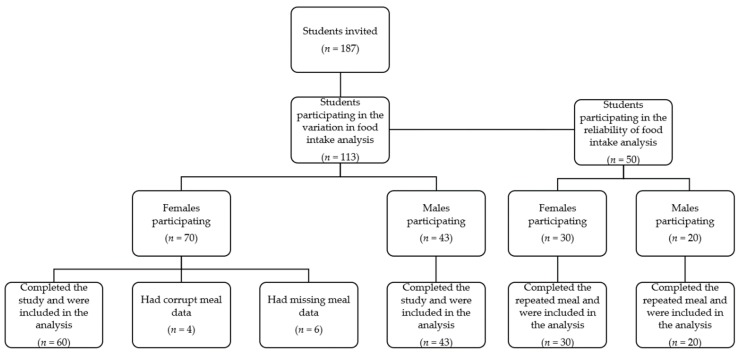
Study diagram.

Six high school classes (*n* = 187) were invited to participate (see Figure 2) in the measures for the variation in food intake analysis. Out of the invited students, 113 accepted to be part of the study, and 103 had complete data, four had corrupt meal data, and six had missing meal data. In all cases the loss of data was caused by errors in the data transmission between the mobile phones (provided to the students for use in the study) and the central data server, partially corrupting or altogether not delivering the appropriate files. All participants with missing and corrupt meal data were females and were excluded from the final data analysis. Out of those with complete data, 60 were females and 43 were males. The study took place in December 2015.

All the students in four out of the six classes previously invited (*n* = 123) were asked to participate in additional meal measurements, for the reliability of food intake analysis (see Figure 2). In total, 50 students (30 females and 20 males) accepted to participate in repeated meal measures. These measurements took place approximately three months (February/March 2016) after the meals for the variation in food intake analysis, and all the students provided complete data.

All invited students were allowed to participate, since no additional inclusion/exclusion criteria existed, other than being part of the invited classes. The participating students were between 15–18 years of age. As a reward, each participating student who completed the study received one cinema ticket per meal.

### 2.4. Data Sources/Measurement

**Figure 3:**
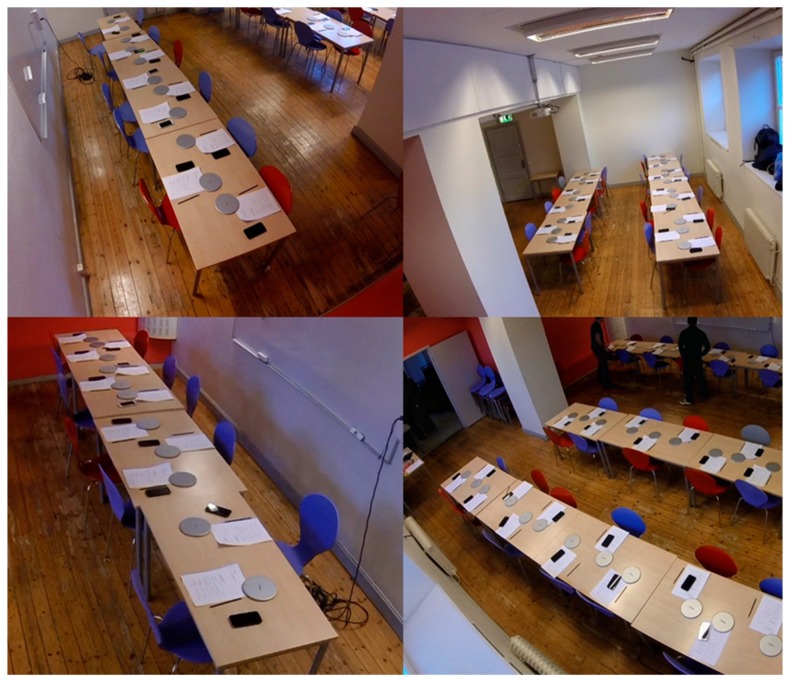
Placement of the cameras recording students during their lunches.

The weight and the height of the students were measured by researchers before the lunches. Immediately before the meal, each student received a digital food scale (Mandometer^©^, Stockholm, Sweden [21]) and a mobile phone. The food scales were used to measure the weight of each student’s food intake, while the mobile phones were used to assess subjective fullness before and after the meal. The food scales were initially placed on the lunch tables (Figure 3) and each student placed their individual plate with food on one of the scales before the initiation of the meal. The plates were then kept on the scales throughout the meal, except during food additions when students were allowed to pick up their plates to add more food from the provided food buffet. The “rate your fullness” scale was presented through the provided mobile, with anchors attached to a 100-point visual analog scale (VAS) from “none” to “extreme”. Another VAS (in paper format) was used to record the student’s subjective evaluation of food taste after the meal (from “extremely bad” to “extremely good”), similar to the methodology previously reported by our group [15]. In addition, the weight scales were connected to the mobile phones through Bluetooth connection and a specifically designed mobile application (created for this specific purpose [20]) was used to upload each student’s meal file to a server through Wi-Fi. Meal files were later downloaded from the server and used for manual analysis of eating behaviors (in combination with video recordings), following a process previously described [22]. 

Four video cameras (GoPro^®^, San Mateo, CA, USA), attached high on the walls/ceiling of the lunch room, were used to record videos of the students while they were eating (Figure 3). One additional camera was positioned above the buffet.

Observer^®^ XT software (Noldus, Wageningen, The Netherlands) was used to annotate all spoonfuls and food additions during the recorded meals. Additionally, the time students spent away from the table in order to revisit the buffet and collect additional food was quantified. Meal duration was calculated as time interval between first and last spoonful minus total time spent away from the table (to collect additional food). Meal duration was used to calculate eating rate as total mass eaten (grams) divided by meal duration (minutes) for each individual student.

### 2.5. Served Food

**Figure 4:**
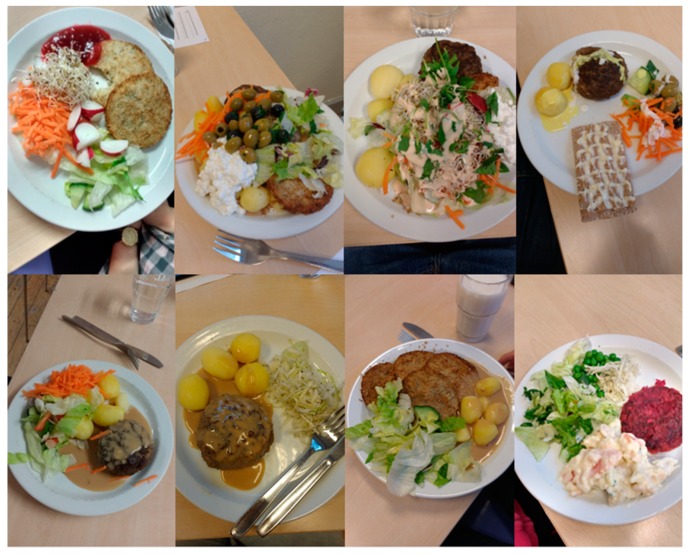
Food choices among a sample of the included students.

The students could freely choose from the following foods during the lunch buffet (see Figure 4 for examples): (1) beef patties made by minced meat, onion, gluten, eggs, salt, and pepper; (2) cream sauce made by onion, garlic, vegetable broth, herbs, milk protein, salt, and pepper; (3) pollock with sauce, which was comprised of pollock, shrimp, milk protein, vegetables, and dill, cooked with white wine; (4) celery patties made by gluten, celery, breadcrumbs, rapeseed oil, boiled potatoes, salt and pepper, and beetroots; (5) side dishes such as cottage cheese, jam, carrots, cucumber, lettuce, olives, sprouts, and crispbread were also available. In addition, students were free to choose either water or milk to drink (not quantified in the current protocol). The offered foods and drinks were identical between the variation in food intake and the reliability of food intake lunches. 

### 2.6. Statistical Methods

The variation in food intake was analyzed using multiple linear regression, with food intake in grams as dependent (response) variable and eating rate (grams eaten/minute), number of spoonfuls, number of food additions, sex, food taste, change in fullness (after meal fullness − before meal fullness), and BMI as independent (explanatory) variables. The independent variables were chosen before conducting the regression analysis. The assumption of normal distribution was checked by visual inspection of normal Q-Q plot, linearity and equal variance by visual inspection of residual plot, collinearity and multicollinearity by variance inflation factor (VIF) and tolerance statistics, and independence by Durbin-Watson statistic. All assumptions of the regression model were fulfilled. Furthermore, a scatter plot of Cook’s distance for each subject was used to evaluate high leverage data points (outliers = observations with Cook’s distance > 4/*n*). One outlier was identified and removed from the model creation.

The reliability of food intake was analyzed based on the following: (1) systematic changes in the mean of repeated meals, (2) absolute reliability (i.e., within-subject random variation expressed as a coefficient of variation (noise)), and (3) relative reliability expressed as a retest intra-class correlation (rank of students in comparison to the group) from one meal to the other [23].

SPSS 24 (IBM, Armonk, NY, USA) software [24] was used for the multiple linear regression model and a publicly available spreadsheet was used for the reliability analysis [25].

### 2.7. Study Size

Based on a sample size of 103 students, an effect size of 0.15 (medium), desired statistical power level of 0.8, and a probability level of 0.05, seven predictor variables could be included in the regression model. G*Power 3.1 was used for power calculation [26].

### 2.8. Ethics Approval and Consent to Participate

All the reported procedures were approved by the Swedish Ethical Review Board (see ethical approval: dnr 2015.1824-31) and followed the requirements of the Helsinki Declaration. Each included student provided the appropriate written consent to participate in the study and for their data to be published as part of the analysis outcomes.

## 3. Results

Table 1 presents the descriptive statistics for the samples of students who participated in the variation in food intake and the reliability of food intake analyses.

### 3.1. Explaining Food Intake

The multiple linear regression model was significant and could explain 76.6% of the variation in food intake among students (see Table 2). The model showed significant associations between food intake and all included predictor variables except change in fullness and BMI. Standardized b coefficients indicated that eating rate was the independent variable with the highest explanatory power in the model (0.537), followed by number of spoonfuls (0.428), sex (0.165), number of food additions (0.160), food taste (0.115), BMI (0.091, not significant), and change in fullness (0.003, not significant). 

### 3.2. Reliability of Food Intake

Means (systematic bias) and standard deviations for food intake, eating rate, number of spoonfuls, number of food additions, food taste, and change in fullness for the two lunch meals and the differences between them are presented in Table 3.

**Figure 5:**
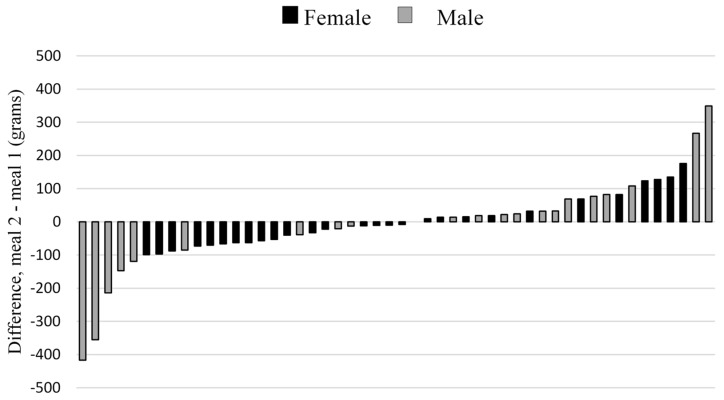
Individual differences in recorded food intake (grams) between two lunches with identical food choices in a Swedish high school (*n* = 50). Black bars: female participants; Grey bars: male participants.

Individual differences between grams of food eaten at the first and the second school lunch ranged from −417 to +349 g (Figure 5).

The typical error expressed as coefficient of variation for food intake was 26.1% (95% CI = 21.4–33.5%) and the intraclass correlation (ICC) coefficient was 0.74 (95% CI = 0.58–0.84).

## 4. Discussion

This is the first study attempting to explain variations in objectively measured food intake in a school lunch setting among high school students and to assess the reliability of food intake in the same context and population.

Eating rate was shown to be the strongest explanatory variable in the regression model. This is in line with previous studies conducted in the school environment [14] and in the laboratory [16] showing the importance of eating rate in determining food intake. Additionally, a randomized controlled trial among obese children showed beneficial effects of modifying the speed of eating on their BMI in comparison to standard lifestyle modification therapy [27]. Since students have only 25 min to eat and collect food in the school included in this study, there is a possibility that the enforced time restriction might push students into faster than normal eating rates, perhaps similar to what Zandian and colleagues observed in younger children [14]. On a related note, when comparing lunch characteristics in the current study against an earlier study that we conducted in the same school (with similar food served) one year earlier [15], students had a 13 g per minute lower eating rate (33 vs. 46 g/min) while their food intake was 24 g lower on average (348 vs. 372 g eaten). Interestingly, during the intervening year, the school had increased the available time for students to eat lunch (25 vs. 20 min), potentially explaining the observed differences. However, it is important to note that the current study was not designed to investigate this difference, and future, properly powered studies need to be conducted to test this hypothesis further, ideally in a within-subject design.

The number of spoonfuls was another powerful explanatory variable for the variation in food intake. For each additional spoonful, an extra 4 g of food intake could be explained by our model. Hermsen and colleagues have shown the potential of a smart fork to decelerate eating rate [28]. Our results suggest that the absolute number of spoonfuls could be another target for reducing food intake during meals, potentially using novel technological tools, such as commercially available smartwatches, to quantify and modify this behavior [29,30].

Previous studies in schools have shown sex differences in food intake in both 12–13-year-old students [14] and 16–17-year-old students [15] that are in line with the current study. However, none of these studies controlled for other factors such as eating rate and BMI. A potential explanation for why females tended to eat less than their male counterparts might be related to differences in sociocultural expectations and elevated self-presentation concerns among the females [31]. Another explanation could be that the female participants had lower energy requirements than the included males (independent of their BMI). However, since we did not measure total energy expenditure among our sample, this point remains speculative. Additionally, past studies examining the observer effect on food intake suggests that women, in general, eat less food when being aware of being observed [31]. For example, a laboratory study that included both males and females found that when participants were aware that their food intake was monitored (observation effect), females ate significantly less food (~8%) while males did not [32].

The explanatory power of food additions is novel. Rolls and colleagues investigated effects of different plate sizes on intake in a buffet setting and observed a non-significant difference in food intake due to increased amount of “trips to the buffet” in the condition with smaller plate size vs. larger [33], but no studies have investigated the direct association between the number of food additions and food intake. Since the model showed that each food addition, independent of other explanatory variables, amounted to ~50 g of extra food intake, interventions targeting food additions in a school lunch buffet setting should be considered. 

The self-reported change in fullness, due to the meal, was not significant in our model. This observation is not surprising since other studies have shown a relatively weak relationship between fullness and food intake and with great variation between studies [18]. On the other hand, subjective food taste was significant, but had only modest explanatory power. 

The reliability analysis showed that food intake is stable on a group level when comparing one school lunch to the other. The systematic bias of these measurements appears to be small and a very low familiarization effect was present [23]. However, on an individual level, many students seem to change their food intake substantially across repeated meals provided in identical settings, with an observed coefficient of variation of ~26% and absolute change ranging from −417 to +349 g. This individual instability in food intake was not confirmed in the intraclass correlation coefficient (0.74) that could be labeled as very large [34]. This can be explained by the large variation in food intake between students [23], which has also been reported before [15]. The high correlation indicates that the rank of students was relatively stable when comparing the two meals. For example, one can consider that a student that eats a lot (with respect to his/her peers) in one school lunch will do the same in a repeated meal, even if the individual variation in food intake is high. Thus, one school lunch could be used to rank most students in comparison to their peers. This observation is in line with our previous study in young adult women eating repeated meals in a laboratory [35].

Our study has several limitations. For the main analysis to explain food intake, a cross-sectional study design was used, allowing us to report parameter associations but not causal relationships. Results obtained from the regression model should therefore be interpreted with caution, especially since we did not validate the model in an independent dataset. As previously shown, habitually eating large portions of food might be a risk factor for increased energy intake and it is an important outcome to investigate [36]. However, in the current setting, since foods with different energy densities were served, we cannot be sure that increased food intake directly translated into higher energy intake [37]. In addition, we did not record or quantify the drink choice of the students during the lunches (limited to either water or milk), further complicating potential calculations of energy intake. This is important since children and adolescents with obesity may need higher amounts of fluid in order to perceive fullness [38]. Similarly, our protocol did not allow us to quantify food leftovers on the plate, a variable of great importance (if combined with the food eaten to allow calculation of portion size) to explain food intake [37]. The students included in the current study had a lower prevalence of overweight and obesity (~12%) vs. a representative sample of the general population of Swedish students attending high school (~24%) [6]. Thus, one should be careful to generalize our outcomes for the whole student population. A potential systematic bias in our results might be the fact that students were eating under observation [31]. However, since no studies have previously been conducted in the school lunch context to investigate the observer effect, this point remains speculative.

Future experimental studies should investigate the effects of lunchtime length (i.e., 20 vs. 30 min allowed in the lunch canteen) on eating rate and subsequent food intake among students. More detailed measures of the amounts (grams) eaten for each specific food type could allow accurate calculations of energy intake and should therefore be pursued in future studies in the school lunch setting. The explanatory power of portion size and energy density on food intake should be investigated.

## 5. Conclusions

Our results illustrate the importance of objectively measured variables such as eating rate, the number of food additions, and the number of spoonfuls in comparison to subjectively rated fullness and food taste to explain variations of food intake in a school lunch setting. Interventions aimed at modifying eating rate, spoonfuls, and/or food additions might be successful in changing students’ FI in this setting. Finally, the methods used in the current study show promise for large-scale quantification of objectively measured food intake and eating behaviors in schools and beyond.

## Figures and Tables

**Table 1:** Characteristics of students in the variation in food intake and reliability of food intake ^1^ analyses ^2^.

	Variation in Food Intake (*n* = 103)	Reliability of Food Intake (*n* = 50)
Age, year	16.7 ± 0.6	16.8 ± 0.6
Female sex, (%)	60 (58%)	30 (60%)
Weight, kg	61.8 ± 12.1	61.2 ± 11.1
Height, cm	170.3 ± 9.5	169.0 ± 9.0
BMI, kg/m^2^	21.2 ± 3.2	21.4 ± 3.1

^1^ Students included in the reliability of food intake analysis were all participating in the variation in food intake analysis. ^2^ Data are presented as mean ± standard deviation. BMI: body mass index.

**Table 2:** Multiple linear regression model ^1^ explaining variations in food intake.

Model	*B*	Lower Bound 95% Confidence Interval for *B*	Upper Bound 95% Confidence Interval for *B*	*t*	*p*
Constant	−212.978	−341.133	−84.823	−3.300	0.001
Eating rate (grams/minute)	5.419	4.276	6.562	9.414	<0.001
Number of spoonfuls	4.143	3.111	5.175	7.969	<0.001
Sex ^2^	58.326	19.824	96.827	3.008	0.003
Number of food additions	48.210	16.570	79.850	3.025	0.003
Food taste	1.159	0.097	2.221	2.167	0.033
BMI	5.008	−0.677	10.693	1.749	0.084
Change in fullness ^3^	0.021	−0.702	0.744	0.058	0.954

^1^ Adjusted R2 0.766 and R2 0.783. Model is significant, *p* < 0.001. Parameters ordered based on explanatory power (standardized b coefficients, see text). ^2^ Sex: 1 = male, 0 = female. ^3^ Change in fullness is calculated as fullness after the meal − fullness before the meal. *B* = unstandardized b coefficients, *t* = the *t* test statistic, *p* = the probability value.

**Table 3:** Mean values of meal parameters in the two lunches and the difference between them ^1^.

	Lunches		Difference (Second − First)
	First (*n* = 50)	Second (*n* = 50)	
Food intake (grams)	351.8 ± 171.0	344.3 ± 171.6	−7.5 ± 125.1
Eating rate (grams/minute)	30.2 ± 15.4	34.6 ± 19.3	4.4 ± 12.9
Number of spoonfuls	42.7 ± 18.8	39.0 ± 16.2	−3.7 ± 15.5
Number of food additions	0.3 ± 0.5	0.1 ± 0.4	−0.2 ± 0.6
Food taste	48.4 ± 15.1	49.0 ± 16.2	0.6 ± 14.9
Change in fullness	33.4 ± 25.2	40.3 ± 32.2	6.9 ± 32.0

^1^ Data are presented as mean ± standard deviation.
